# Biosurfactant: A Next-Generation Tool for Sustainable Remediation of Organic Pollutants

**DOI:** 10.3389/fmicb.2021.821531

**Published:** 2022-02-21

**Authors:** Neha Sharma, Meeta Lavania, Banwari Lal

**Affiliations:** Microbial Biotechnology, Environmental and Industrial Biotechnology Division, The Energy and Resources Institute (TERI), New Delhi, India

**Keywords:** organic pollutants, biosurfactant, bioremediation, environment, polluant

## Abstract

Petroleum hydrocarbons are energy resources that majorly contribute pollutants to the environment. These pollutants may cause serious health issues, and hence, for the regulation of these contaminants, the development of sustainable alternative technologies has been considered, without causing further harm to the environment. One such alternative is biosurfactants (having low toxicity and being biodegradable) produced by numerous microbial species that have a tendency to remediate organic pollutants. Biosurfactants are amphiphilic compounds that are categorized into two types based on their molecular mass. Biosurfactants can be generated extracellularly or as a part of the cell membrane of microorganisms (bacteria, fungi, and algae). This review provides a detailed view of the types of biosurfactants, their properties, and the mechanism involved in the degradation of oil spills.

## Introduction

**FIGURE 1:**
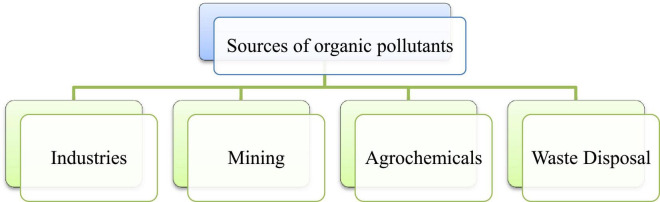
Various sources of organic pollutants impacting the environment.

Organic compounds are utilized extensively in the industrial and agricultural sectors (Bhatt et al., 2021). Rapid industrialization has increased the chances of environmental contamination with numerous organic pollutants, *viz.*, benzene, chloroform, gasoline, plastic compounds, pesticides, and paints (Chen and Chang, 2020). Organic pollutants are ubiquitously present in the ecosystem such as air, soil, and water (Figure 1). These pollutants have harmful effects on human health and the flora and fauna found in the ecosystem (Bhatt et al., 2019).

Various reports are available on the impact of organic pollutants, which appear as carcinogens and mutagens in nature, on human health (Yoshikawa et al., 2017). However, the remediation strategies for such harmful pollutants are crucial. Few remediation methods are implemented such as soil washing, pumping, aeration, oxidation, and incineration (Yoshikawa et al., 2017). These remediation methods have various drawbacks including the development of other secondary contaminants which are uneconomical. Therefore, to mitigate such problems, microbial bioremediation is considered as one of the economical and sustainable approaches for the remediation of environmental contaminants (Bhatt et al., 2019).

The microbial bioremediation of pollutants involves enzymatic reactions carried out by microbial species that lead to the generation of intermediate metabolites (glycolipid). This glycolipid plays an efficient role in the uptake of such pollutants into the microbial membranes (Liston et al., 2017). Microbial glycolipids act as emulsifiers and are known as “biosurfactants”, produced by various microbial species (Chong and Li, 2017).

During microbial growth, a range of compounds was used to drive energy and as a carbon source. However, the carbon form found in hydrocarbons in insoluble form and various microorganisms (yeast and bacteria) diffuse through biosurfactants. Biosurfactants play a critical role in emulsifying the hydrocarbons in the medium (Leuchtle et al., 2015). For example, different species of *Pseudomonas* produce rhamnolipids with a surface tension value of 29 mN/m. Most microorganisms altered their cell wall due to the production of lipopolysaccharides in the cell wall (Saenz-Marta et al., 2015).

### Structures and Role of Biosurfactant

**FIGURE 2:**
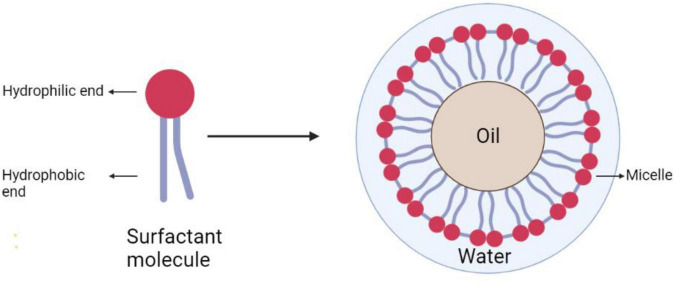
Schematic representation of surfactant and its behavior at the water–oil interface, leading to micelle formation.

Surfactant molecules consist of two ends, *viz.*, hydrophilic and hydrophobic ends, as shown in Figure 2. The hydrophilic region is water-soluble, which may include carbohydrate, amino acid, cyclic protein peptide, phosphate, or alcohol, whereas the hydrophobic region is made up of hydrocarbons consisting of long-chain fatty acids, hydroxyl fatty acids, or α-alkyl-β-hydroxy fatty acids, which are mainly found at the C8 to C22 alkyl chains or alkyl aryls (Rahman and Gakpe, 2008). The amphiphilic moiety of surfactants is efficient in reducing the surface and interfacial tension between the individual molecules at both the interface and surface (Maikudi Usman et al., 2016).

The surfactants are extensively used as they increase the surface area and bioavailability of the hydrophobic organic substrates, while controlling the proliferation of microbial biofilm formation onto the surfaces (Vijayakumar and Saravanan, 2015). The surfactant in the mixture of water and oil will lie at the water–oil interface, which provided foaming, detergency, emulsifying, and dispersing capacities that make it a suitable material for remediation (Saenz-Marta et al., 2015).

#### Stratification of Biosurfactants

The classification of biosurfactants majorly depended on the origin of the microbes and their chemical composition. Biosurfactants are divided into two types based on molecular weight: low-molecular-weight compounds that have lower interfacial surface tension and high-molecular-weight biosurfactants that are the most efficient stabilizing agents. The low-molecular-weight biosurfactants are glycolipids, lipopeptides, and phospholipids, whereas polymeric surfactants come under the category of high-molecular-weight biosurfactants (Table 1).

**TABLE 1:** Diverse types of biosurfactants used for the remediation of organic pollutants.

Type of surfactant	Properties	Microorganisms	Surface tension (mN/m)	Technique for identification	References
Glycolipid	Long-chain aliphatic acids or hydroxyaliphatic acids linked through ester groups	Rhamnolipid produced by *Pseudomonas aeruginosa*, trehalolipids produced by *Rhodococcus erythropolis*	29	FTIR, TLC, LC-MS for trehalolipids	Abbasi et al., 2012
Lipopeptides	Lipid attached to the polypeptide chain	*Bacillus licheniformis*	27	FTIR, TLC	Li et al., 2016
Phospholipids	Lipid associated with the phosphate group	*Klebsiella pneumoniae* WME02		Biochemical characterizations	Jamal et al., 2012
Polymeric biosurfactants	Polysaccharides associated with protein complex, including emulsan, liposan, and lipomannan	Emulsan was produced by *Acinetobacter calcoaceticus*; liposan was produced by *Candida lipolytica*	29–32	TLC, HPLC-ESI-MS	Anaukwu et al., 2015; Santos et al., 2017

### Properties of Biosurfactants

**FIGURE 3:**
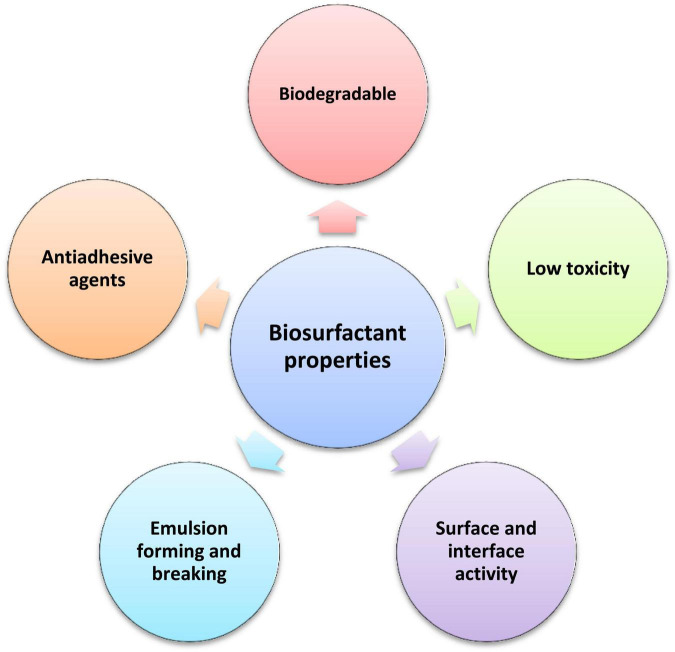
Highlighting the properties of biosurfactants as an economical approach for remediation of pollutants.

Unique and distinct properties of biosurfactants that make them sustainable alternatives over their chemically synthesized counterparts are as follows (Figure 3).

#### Surface and Interface Activity

The systematic force between liquid molecules is known as surface tension. The indistinguishable condition also applies to the interface between two immiscible liquids, such as oil in water, which is identified as interfacial tension (IFT). The tendency of a microbial surfactant is determined by its stability to downregulate the surface tension of the media. An efficient biosurfactant is capable of lowering the surface tension of water.

Some reports clearly showed that *Bacillus subtilis* produces surfactin that effectively reduces the surface tension under harsh conditions. Another study showed that rhamnolipid, a biosurfactant produced by *Pseudomonas aeruginosa*, significantly decreases water surface tension as compared to the other surfactants (Kim et al., 2015). A report by Mulligan (2005) showed the efficient role of microbial surfactants in decreasing the surface tension of water from 72.0 to 35.0 mN/m, which reflects the potential of biosurfactants.

### Biodegradability

Another interesting property of biosurfactants is their degradability. Biosurfactants are non-toxic and non-hazardous material, making them suitable for application in various industries such as cosmetic, food, and pharmaceutical. A study showed that the LC50 of emulsan against *Photobacterium phosphoreum* is significantly lesser than against *Pseudomonas rhamnolipids* (Gregorich et al., 2015).

The biodegradability test of sophorolipid biosurfactants produced by the non-pathogenic yeast *Candida bombicola*, conducted in accordance with the OECD Guidelines for Chemical Testing (301C Modified MITI test), showed that biodegradation of biosurfactants begins shortly after cultivation. Biodegradability is also expressed in the form of biochemical oxygen demand (BOD)/theoretical oxygen demand (TOD). The ratio of the required amount of sophorolipid to the total required amount of oxygen achieved after 8 days of culture was at the 61% level. The other two biosurfactants studied (surfactin and arslofactin) were also degraded as sophorolipids (Hirata et al., 2009).

#### Anti-adhesive Property

Pre-adhesion of biosurfactants to solid surfaces provides a new and effective means of combating colonization by pathogenic microorganisms, as biosurfactants inhibit the attachment of pathogenic organisms to solid surfaces or sites of infection (Singh and Cameotra, 2004). The biosurfactants can also be used in modifying the hydrophobicity of the surface that directly affects the adhered microbial population forming a biofilm. A report showed that biosurfactants from *Pseudomonas fluorescens* inhibited the attachment of *Listeria monocytogenes* onto steel surfaces (Chakrabarti, 2012).

The literature demonstrates the anti-adhesive activity of crude biosurfactants against most of the microorganisms even in the minimum concentration of 0.75 mg/L. There is a direct correlation between the anti-adhesive property and the concentration of the biosurfactant. An anti-adhesive specificity was detected against *Lactobacillus casei*, with values of 91 and 99% at minimal biosurfactant concentration (0.75 mg/L). Low inhibitions were observed for *Staphylococcus epidermidis* and *Escherichia coli*, with values of 27 and 21%, respectively, at maximum biosurfactant concentration (Diniz et al., 2013).

#### Emulsion Forming and Emulsion Breaking

Biosurfactants may act as emulsifiers or de-emulsifiers. An emulsion can be a heterogeneous system, comprising one immiscible liquid dispersed in another in the form of droplets. There are two types of emulsions: oil-in-water (o/w) or water-in-oil (w/o) emulsions. These emulsions are not stable and were thus stabilized with the addition of biosurfactants. *Candida lipolytica* produce water-soluble liposan, which was used to emulsify edible oils by coating droplets of oil, thus forming stable emulsions (Vijayakumar and Saravanan, 2015).

The report showed that the production of biosurfactants from *P. aeruginosa* RB 28 started at the late log phase and reached its maximal level (2.7 g/L) at the stationary phase. The produced rhamnolipid showed the efficient emulsification of sunflower oil, heptadecane, and paraffin. Biosurfactants showed stable emulsion formation with hydrocarbons (Sifour et al., 2007).

#### Mechanism of Bioremediation Induced by Biosurfactant

**FIGURE 4:**
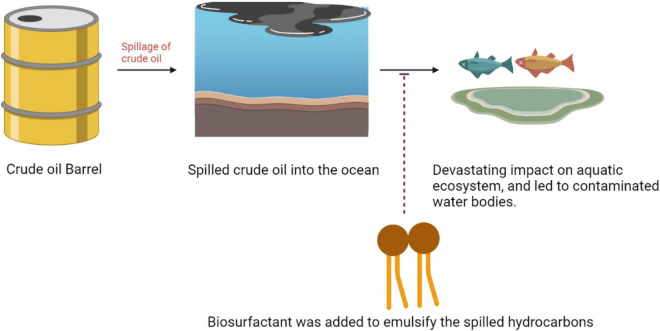
Adverse effects of oil spilling and solution to mitigate such problems.

The oil spills cause a devastating impact on oceanic life in marine climate. To mitigate spilled oil, artificially synthesized chemical surfactants had been considered in the ocean, but there are various drawbacks associated with them such as toxicity and degradability. One of the intrinsic choices for this purpose was to identify the biomolecules which had surface activity along with the emulsifying action (Figure 4). When the biosurfactants are released in a water–oil suspension, their monomers organize in sphere-forming micelles in such a way that the hydrophobic end of the biosurfactant is turned to the center, composing the nucleus, and the hydrophilic part is turned to the sphere surface, making an interface with the water. Therefore, the surfactant molecule reduces the surface tension between the water and oil interfaces and increases the hydrocarbon exposure to the bacteria and oxygen, facilitating hydrocarbon biodegradation (Soberon-Chavez and Maier, 2010). The alterations in the membrane such as modification in the protein composition or increase in hydrophobicity of the cell wall caused by biosurfactants promote higher accessibility of the hydrocarbons to the microorganisms. The aggregates were generated *via* weak chemical interactions such as Van der Waals and hydrogen bridges. Micelle formation leads to the decline in the force of repulsion between the immiscible liquid phases (Aparna et al., 2012).

The biosurfactants emulsify the hydrocarbons in water to shape different blends and make them water-soluble. Lichenysins, rhamnolipids, and surfactin are some of the surfactants that are viewed as effective in the remediation of oil defilement (Peele et al., 2018). Literature proposed that biosurfactants developed by the marine bacterium were sufficiently able to obliterate the oil spills which float on the outer layer of water to advance the scattering of oil in water by shaping a steady emulsion, in this way upgrading the pace of biodegradation. Due to these variables, biosurfactants had shown potential in tidying up oil slicks on coastlines and in the ocean. There is a pervasive presence of marine microorganisms which debase hydrocarbons, which have been perceived as hydrocarbonoclastic microbes. These microbes degrade the hydrocarbons present in the polluted sites of marine environments. Various investigations demonstrate that the combination of the biosurfactants stimulated the degradation of hydrocarbons in the marine climate. A hydrocarbonoclastic bacterial consortium has a wide scope of degradation capacities on both aliphatic and aromatic fractions of crude petroleum oil. As a rule, biosurfactants delivered by oil-degrading microorganisms can upgrade the absorption of the hydrocarbons just as the supplements accessible in the climate (Peele et al., 2018).

Few microorganisms develop emulsifying agents that could help in hydrocarbon degradation; subsequently, emulsifiers have been utilized for tidying up oil (Bach et al., 2003). Biosurfactants can be scaled up to industrial scale by the fermentation process. Lichenysins were delivered from *Bacillus licheniformis* JF-2, which was extracted from well water. Lichenysins even at lower concentrations (10–60 mg/L) are able to diminish the surface pressure between interfacial surfaces into much lower levels (10^–2^ mN/m). The range of temperature (≤140°C), pH (6–10), and salinity (up to 10% w/v NaCl) deviation had no impact on their activity (Bach et al., 2003).

The knowledge of utilizing biosurfactant systems for improved remediation of oil spills is growing; and biosurfactants which are bio-based and biodegradable could potentially solve issues related to oil spillage. Furthermore, the need for advancing the understanding of mechanisms between oil spills and microbial surfactants still exists.

#### Commercial Biosurfactants for MEOR Application

The biosurfactants can be manufactured using renewable resources and waste products that make the overall process economical (Rufino et al., 2014). Globally, biosurfactant manufacturers include Jeneil Biotech, Ecover, Soliance, Saraya, MG Intobio, and AGAE Technologies, occupying the markets of North America, Europe, and Asia-Pacific (Sajna et al., 2015). Among all the companies, the most achievable effort was carried out by Jeneil Biosurfactant Co., (Saukville, WI, United States) who has successfully scaled up the development of biosurfactant up to a batch of 20,000 gallons (Rufino et al., 2014).

According to reported studies, the global market for these “green” alternative biosurfactants compared with synthetic surfactants reached US$1735.5 million in 2011. In 2013, the total production was approximately 344 kilotons. The annual average growth rate is expected to reach 4.3% during 2014–2020 (Gudina et al., 2015; Grand View Research, 2016).

## Conclusion

Microbial biosurfactants are broadly investigated for bioremediation purposes, and numerous investigations have confirmed the degradation-specific role of biosurfactants. Biosurfactants play a key role in the adhesion of cells in biofilms that increase the degradation efficiency for organic pollutants. Biosurfactants typically display efficient micelle formation when compared with synthetic surfactants and are biodegradable with various biological activities. Thus, they can have exciting applications in various industries such as food processing, cosmetics preparation, and pharmaceutical additives. Hence, recent advances in microbial biosurfactants have been added to their significant applications in various other industries. There is a need to explore and understand the mechanism involved in the remediation of organic pollutants.

## Author Contributions

NS and ML conceived and designed the experiments. All authors drafted the work, revised it critically for important intellectual content, and approved the final version to be published.

## Conflict of Interest

NS was employed by ML and BL. All authors declare that the research was conducted in the absence of any commercial or financial relationships that could be construed as a potential conflict of interest.

## Publisher’s Note

All claims expressed in this article are solely those of the authors and do not necessarily represent those of their affiliated organizations, or those of the publisher, the editors and the reviewers. Any product that may be evaluated in this article, or claim that may be made by its manufacturer, is not guaranteed or endorsed by the publisher.
